# Supplementary material for: Quick model-based viscoelastic clot strength predictions from blood protein concentrations for cybermedical coagulation control
Source: Nat Commun. 2024 Jan 5;15:314. doi: 10.1038/s41467-023-44231-w (PMC10770315; doi:10.1038/s41467-023-44231-w)
Supplement: Supplementary file 1 — Supplementary Information [file 41467_2023_44231_MOESM1_ESM.pdf]

Quick Model-Based Viscoelastic Clot Strength Predictions from Blood Protein Concentrations for Cybermedical Coagulation Control

Supplementary Information

Damon E. Ghetmiri[1,2], Alessia J. Venturi[1], Mitchell J. Cohen[3,4], and Amor A. Menezes[1,5,6,7*]

[1] Department of Mechanical and Aerospace Engineering, University of Florida, 527 Gale Lemerand Drive, Gainesville, FL 32611-6250, USA.
[2] Now at: ASML, 17075 Thornmint Court, San Diego, CA 92127-2413, USA.
[3] Department of Surgery, University of Colorado Denver, 12631 East 17th Avenue, Mailstop C305, Aurora, CO 80045-2527, USA.
[4] Center for Combat Medicine and Battlefield (COMBAT) Research, Department of Emergency Medicine, University of Colorado Denver, 12401 East 17th Avenue, Mailstop B215, Aurora, CO 80045-2589, USA.
[5] J. Crayton Pruitt Family Department of Biomedical Engineering, University of Florida, 1275 Center Drive, Gainesville, FL 32611-6131, USA.
[6] Department of Agricultural and Biological Engineering, University of Florida, 1741 Museum Road, Gainesville, FL 32611-0570, USA.
[7] Genetics Institute, University of Florida, 2033 Mowry Road, Gainesville, FL 32610-3610, USA.
[*] Corresponding author: amormenezes@ufl.edu

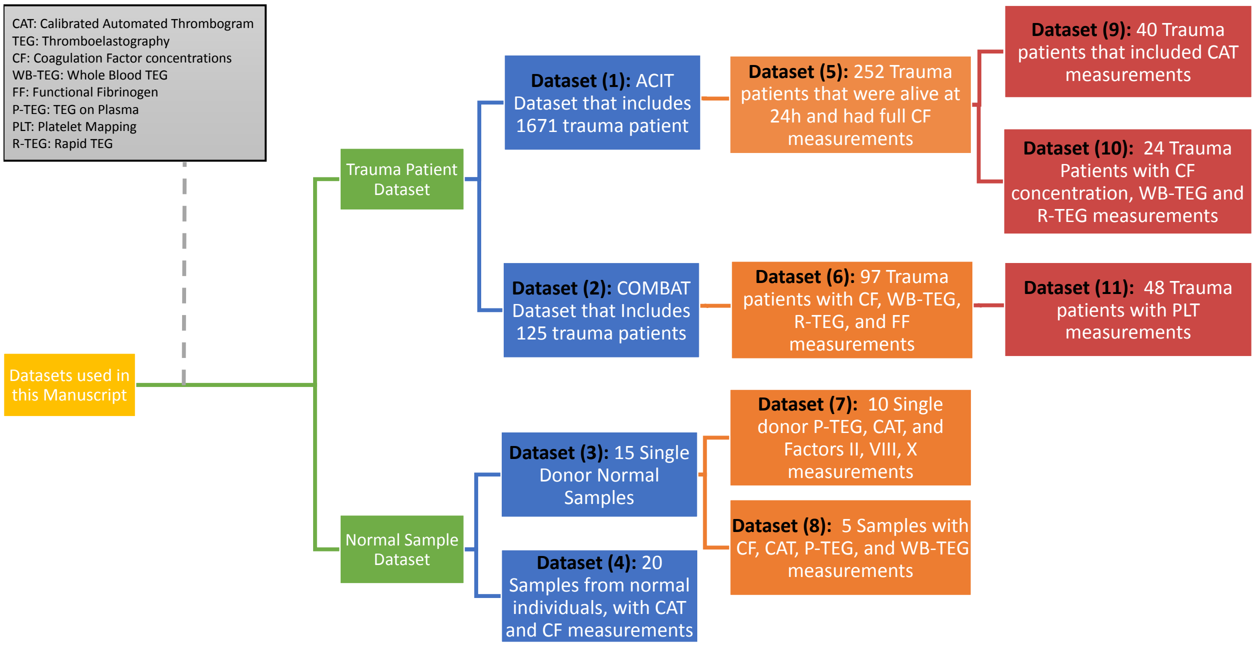

Objective	Content	Dataset Used
Viscoelastic model of plasma clot strength	Fig. 4	(7)
Five-fold cross validation of plasma viscoelastic model	Fig. 4	(7)
Validation of plasma viscoelastic model	Fig. 5	(8)
Functional fibrinogen level quantification	Fig. 5	(6)
Viscoelastic model of whole blood clot strength	Fig. 6	(10)
Five-fold cross validation of whole blood viscoelastic model	Fig. 6	(10)
Validation of whole blood viscoelastic model	Fig. 7	(8)
Correlation between whole blood model and TEG Citrated Native assay	Fig. 7	(10)
Platelet effect quantification	Fig. 8	(11)
Correlation between TEG Citrated Native and Rapid TEG assays	Fig. 9	(6)

Supplementary Figure 1: Dataset overview and summary. Datasets used for the different model development, analysis, and simulation results in this article. As indicated, separate datasets were used for training and validation. The Activation of Coagulation and Inflammation in Trauma (ACIT) study[1] was a single-center prospective cohort study that followed trauma patients from emergency department admission through discharge from hospitalization or death. The Control of Major Bleeding After Trauma (COMBAT) study[2] was a single-center prospective randomized controlled trial that included TEG assays (Functional Fibrinogen, Citrated Native, Platelet Mapping, and Rapid TEG) on trauma patient blood samples.

Dataset (6) Trauma Patient Characteristics

Characteristic	Mean ± std. dev. or percentage (no. out of 97)
Age	36.3 ± 13.6
Male / female	79.4% (77) / 20.6% (20)
NISS	27.9 ± 19.7
Blunt / penetrating injury	51.5% (50) / 50.5% (49)
Alive / dead	88.7% (86) / 11.3% (11)

Dataset (10) Trauma Patient Characteristics

Characteristic	Mean ± std. dev. or percentage (no. out of 24)
Age	49.5 ± 23.0
Male / female	83.3% (20) / 16.7% (4)
ISS	27.5 ± 15.5
Blunt / penetrating injury	91.7% (22) / 8.3% (2)
TBI not present / present	16.7% (4) / 83.3% (20)
Alive / dead	70.8% (17) / 29.2% (7)

Dataset (11) Trauma Patient Characteristics

Characteristic	Mean ± std. dev. or percentage (no. out of 48)
Age	36.5 ± 14.0
Male / female	79.2% (38) / 20.8% (10)
NISS	29.0 ± 19.4
Blunt / penetrating injury	43.8% (21) / 58.3% (28)
Alive / dead	87.5% (42) / 12.5% (6)

Supplementary Figure 2: Summary of patient characteristics. Demographic and injury characteristics for Datasets (6), (10), and (11), Supplementary Fig. 1. The COMBAT dataset used the new injury severity score (NISS), while the ACIT dataset used the injury severity score (ISS). Three patients had both a penetrating and blunt injury across the three datasets, two in Dataset (6) and one in Dataset (11); these patients are counted in both categories.

References

1. Cohen MJ, Bir N, Rahn P, Dotson R, Brohi K, Chesebro BB, et al. Protein C depletion early after trauma increases the risk of ventilator-associated pneumonia. Journal of Trauma and Acute Care Surgery. 2009;67(6):1176–1181.

2. Moore HB, Moore EE, Chapman MP, McVaney K, Bryskiewicz G, Blechar R, et al. Plasma-first resuscitation to treat haemorrhagic shock during emergency ground transportation in an urban area: a randomised trial. The Lancet. 2018;392(10144):283–291.
