# Supplementary material for: Quick model-based viscoelastic clot strength predictions from blood protein concentrations for cybermedical coagulation control
Source: Nat Commun. 2024 Jan 5;15:314. doi: 10.1038/s41467-023-44231-w (PMC10770315; doi:10.1038/s41467-023-44231-w)
Supplement: Supplementary file 3 — Reporting Summary [file 41467_2023_44231_MOESM3_ESM.pdf]

nature portfolio

Corresponding author(s): Amor A. Menezes

Last updated by author(s): Nov 30, 2023

Reporting Summary

Nature Portfolio wishes to improve the reproducibility of the work that we publish. This form provides structure for consistency and transparency in reporting. For further information on Nature Portfolio policies, see our Editorial Policies and the Editorial Policy Checklist.

Statistics

For all statistical analyses, confirm that the following items are present in the figure legend, table legend, main text, or Methods section.

n/a	Confirmed	
[ ]	[x]	The exact sample size (*n*) for each experimental group/condition, given as a discrete number and unit of measurement
[ ]	[x]	A statement on whether measurements were taken from distinct samples or whether the same sample was measured repeatedly
[x]	[ ]	The statistical test(s) used AND whether they are one- or two-sided<br>*Only common tests should be described solely by name; describe more complex techniques in the Methods section.*
[x]	[ ]	A description of all covariates tested
[x]	[ ]	A description of any assumptions or corrections, such as tests of normality and adjustment for multiple comparisons
[ ]	[x]	A full description of the statistical parameters including central tendency (e.g. means) or other basic estimates (e.g. regression coefficient) AND variation (e.g. standard deviation) or associated estimates of uncertainty (e.g. confidence intervals)
[x]	[ ]	For null hypothesis testing, the test statistic (e.g. *F*, *t*, *r*) with confidence intervals, effect sizes, degrees of freedom and *P* value noted<br>*Give P values as exact values whenever suitable.*
[x]	[ ]	For Bayesian analysis, information on the choice of priors and Markov chain Monte Carlo settings
[x]	[ ]	For hierarchical and complex designs, identification of the appropriate level for tests and full reporting of outcomes
[x]	[ ]	Estimates of effect sizes (e.g. Cohen's *d*, Pearson's *r*), indicating how they were calculated

Our web collection on statistics for biologists contains articles on many of the points above.

Software and code

Policy information about availability of computer code

Data collection	The MATLAB code and data it uses are available at: https://github.com/SYBORGS-Lab/Viscoelastic-Clot-Model
Data analysis	Data analysis was performed using a contemporary desktop running MATLAB R2021a on a quadcore Intel Core i7-4790 at 3.40GHz with 16GB RAM. Model parameters were fit to experimental data using MATLAB SImulink Design Optimization (SDO) toolbox version 3.9.1.

For manuscripts utilizing custom algorithms or software that are central to the research but not yet described in published literature, software must be made available to editors and reviewers. We strongly encourage code deposition in a community repository (e.g. GitHub). See the Nature Portfolio guidelines for submitting code & software for further information.

Data

Policy information about availability of data

All manuscripts must include a data availability statement. This statement should provide the following information, where applicable:

- Accession codes, unique identifiers, or web links for publicly available datasets
- A description of any restrictions on data availability
- For clinical datasets or third party data, please ensure that the statement adheres to our policy

Source data are provided with this article. Datasets are available at: https://github.com/SYBORGS-Lab/Viscoelastic-Clot-Model

Research involving human participants, their data, or biological material

Policy information about studies with human participants or human data. See also policy information about sex, gender (identity/presentation), and sexual orientation and race, ethnicity and racism.

Reporting on sex and gender	Activation of Coagulation and Inflammation in Trauma (ACIT) study: 1,671 trauma patients of which 1,367 were male (81.45%).<br>Control of Major Bleeding After Trauma (COMBAT) study: 144 trauma patients were enrolled into the study, with as-treated analyses for 125 trauma patients of which 103 were male (82.4%).
Reporting on race, ethnicity, or other socially relevant groupings	Patient characteristics are in Supplementary Fig. 2.
Population characteristics	Patient characteristics are in Supplementary Fig. 2.<br>ACIT study: age 41.0 ± 18.6, ISS 17.7 ± 15.6<br>COMBAT study: age 36.5 ± 13.9, NISS 27.0 ± 19.4
Recruitment	ACIT study: Trauma patients meeting criteria for highest triage activation level were enrolled. Exclusion criteria included patient age less than 15 years, pregnancy, incarceration, and transfer from outside hospital.<br>COMBAT study: Trauma patients undergoing rapid ground transport to an urban Level I trauma center were enrolled. Exclusion criteria included patient age less than 18 years, pregnancy, incarceration, and lack of consent.
Ethics oversight	Normal platelet-poor plasma samples were purchased from Precision BioLogic, Dartmouth, Nova Scotia, Canada. Normal whole blood samples were purchased from Innovative Research, Novi, Michigan, US.<br>ACIT study: University of California Institutional Review Board (reference number 10-04417).<br>COMBAT study: Colorado Multiple Institutional Review Board (reference number 12-1349).

Note that full information on the approval of the study protocol must also be provided in the manuscript.

Field-specific reporting

Please select the one below that is the best fit for your research. If you are not sure, read the appropriate sections before making your selection.

☒ Life sciences ☐ Behavioural & social sciences ☐ Ecological, evolutionary & environmental sciences

For a reference copy of the document with all sections, see nature.com/documents/nr-reporting-summary-flat.pdf

Life sciences study design

All studies must disclose on these points even when the disclosure is negative.

Sample size	For the ACIT study, between February 2005 and May 2016, 1,671 trauma patients meeting criteria for highest triage activation level were enrolled into the study. For the COMBAT study, between April 2014 and March 2017, 144 trauma patients were enrolled into the study, with as-treated analyses for 125 trauma patients. All available patient data was harnessed for maximum model predictive power.
Data exclusions	No data were excluded. Patient data were grouped into subsets and used according to the amount of information contained in each subset.
Replication	External validation of models involved the use of at least five biological replicates, as described in the manuscript.
Randomization	There was no randomization. Model predictions were both cross-validated and externally validated.
Blinding	Since this work constitutes secondary analysis of existing research data, the investigators were blinded to trauma patient data collection.

Reporting for specific materials, systems and methods

We require information from authors about some types of materials, experimental systems and methods used in many studies. Here, indicate whether each material, system or method listed is relevant to your study. If you are not sure if a list item applies to your research, read the appropriate section before selecting a response.

Materials & experimental systems

n/a	Involved in the study
☒	☐ Antibodies
☒	☐ Eukaryotic cell lines
☒	☐ Palaeontology and archaeology
☒	☐ Animals and other organisms
☐	☒ Clinical data
☒	☐ Dual use research of concern
☒	☐ Plants

Methods

n/a	Involved in the study
☒	☐ ChIP-seq
☒	☐ Flow cytometry
☒	☐ MRI-based neuroimaging

Clinical data

Policy information about clinical studies

All manuscripts should comply with the ICMJEguidelines for publication of clinical research and a completedCONSORT checklist must be included with all submissions.

Clinical trial registration	NCT01838863
Study protocol	Reference 60
Data collection	Between April 2014 and March 2017, 144 trauma patients were enrolled into the study, with as-treated analyses for 125 trauma patients.
Outcomes	For more detail, see Supplementary Fig. 1.<br>Dataset (6): 97 trauma patients with coagulation factor concentrations, whole blood TEG, Rapid TEG, and Functional Fibrinogen measurements.<br>Dataset (11): 48 trauma patients with Platelet Mapping measurements.
